# Post-rehabilitation programme to support upper limb recovery in community-dwelling stroke survivors: a mixed methods cluster-feasibility controlled trial

**DOI:** 10.1136/bmjopen-2024-088301

**Published:** 2024-10-15

**Authors:** Katy Pedlow, Niamh C Kennedy, Natalie Klempel, Janice J Eng, Gary Adamson, Jenny Hylands, Noelene Hughes, Zoe Campbell, Suzanne McDonough

**Affiliations:** 1 Ulster University, Belfast, UK; 2 Department of Physical Therapy, University of British Columbia, Vancouver, British Columbia, Canada; 3 Northern Ireland Chest Heart and Stroke, Belfast, UK; 4 Physiotherapy, Royal College of Surgeons in Ireland Faculty of Medicine and Health Sciences, Dublin, Ireland

**Keywords:** Stroke, REHABILITATION MEDICINE, Community-Based Participatory Research

## Abstract

**Background:**

Less than 50% of stroke survivors regain their pre-stroke level of upper limb function, compounded with a lack of long-term rehabilitation options available. The Graded Repetitive Arm Supplementary Programme (GRASP) is an evidence-based upper limb programme delivered as a standalone programme to stroke survivors. To improve access to such a programme, there is the potential to combine it with a high-utility community-based exercise programme, such as the post-rehabilitation enablement programme (PREP). We aimed to establish if this was feasible to deliver alongside the experience of stroke survivors and therapists, identify any refinements the intervention and the acceptability of the intervention and trial procedures.

**Methods:**

A cluster feasibility-controlled trial was conducted using both quantitative and qualitative outcome measures with stroke survivors who were discharged from NHS care. Participants completed PREP for 6 weeks (control), with the intervention group also completing GRASP. The GRASP intervention was refined in between five iterative testing cycles. Focus groups with participants explored the acceptability and feasibility. Individual interviews with intervention therapists explored how feasible it was to embed the intervention into practice, and determine the feasibility of a future larger, mixed methods, randomised controlled trial. Clinical endpoints for upper limb and overall function were explored through the Rating of Everyday Arm use in the Community and Home, 10-metre walk test (10MWT) and quality of life via the Shortened Edinburgh Warwick questionnaire. No further suggestions for intervention design were noted after cycle 4.

**Results:**

Recruitment (n=72) and retention levels (84.7%) were high with 61 participants (mean age of 66 years and 49 weeks post-stroke) completing the study. Participants and therapists reported positive acceptability of the intervention with goal setting and family support noted as beneficial. The home exercise programme was noted as challenging. Participants within both groups demonstrated improvements in clinical measures, with the intervention group demonstrating a greater improvement within the Rating of Everyday Arm-use in the Community and Home and the 10MWT.

**Conclusion:**

This study successfully recruited and retained stroke survivors into an upper limb community-based programme. It poses a feasible delivery mechanism to combine evidence-based upper limb approaches with established physical activity programmes in a future large scale and fully powered study.

**Trial registration number:**

NCT05090163.

**STRENGTHS AND LIMITATIONS OF THIS STUDY**

- Recruitment and retention rates were high, demonstrating high acceptability of the study parameters and intervention from the perspectives of stroke survivors.
- There was a greater improvement across outcome measures in the intervention group than in the control group for upper limb function and mobility measures.
- COVID-19 reduced the number of sites available to deliver the intervention and therefore randomisation could not occur, instead, site allocation reflected resource availability.
- The initial intervention design was challenging for participants as it prevented socialisation with the group. Future iterations addressed this resulting in a positive response from participants.

## Background

 Worldwide over 5 million survivors of stroke, are left permanently disabled^1^ with many having difficulties with their upper limb^1^,^3^ and less than 50% of stroke survivors regaining their pre-stroke level of function.^4 5^ Despite this, the rehabilitation provided for the upper limb falls short of the recommended dose.^6^ Intensive rehabilitation that is repetitive and task-orientated is vital to promote recovery^7^,^9^ however, this needs to continue beyond discharge from hospital and community services.^7 10^ The recently updated stroke guidelines recommend stroke survivors receive repetitive task training to support upper limb recovery when movement difficulties are ongoing.^11^ One option to support this is community-based group interventions,^12^,^14^ which allows more survivors to access rehabilitation while being less resource intensive.^4^ There is a range of evidence-based upper limb interventions that have been suggested as demonstrating effectiveness in improving upper limb function and activities of daily living.^11 15^ However, despite stroke survivors highlighting the impact upper limb impairments have on their lives^8^ there is poor implementation of upper limb interventions into rehabilitation in posthospital settings. Two recent meta-analyses highlighted the positive outcome of interventions such as virtual reality and task-orientated practice combined with electrical stimulation on upper limb function^16^ however, these are interventions using costly technology can be challenging to deliver in a community-based group setting. Another issue highlighted with implementation of upper limb interventions includes uncertainty around optimal dose with low amounts of therapy reported which may reduce opportunity to optimise neuroplastic principles.^17^ Therefore, a low-cost, low-therapist resource upper limb intervention that had potential for high repetition, independent practice to increase dose may allow for greater implementation in the community to help continue stroke survivors upper limb recovery post-acute setting.^17^

The Graded Repetitive Arm Supplementary Programme (GRASP) is one example of an upper limb programme that has been delivered successfully to stroke survivors living in the community setting. Designed to be completed with minimal resources,^14^ the programme focuses on five aspects of upper limb function including fine motor skills completed as part of a home exercise programme (HEP). Participants are then supported to discuss, and problem solve their experience of completing the HEP in subsequent sessions. The programme is grounded by neuroplasticity principles and behaviour change aspects similar to constraint induced movement therapy, including high repetition, goal setting and education.^1214 18^,^21^ The lack of constraint may make GRASP more amenable to community dwelling adults alongside the ability to complete GRASP independently. GRASP has been used in several settings, including virtually, inpatient/outpatient hospitals, as well as community rehabilitation groups.^1322^,^28^ GRASP has traditionally been delivered as a stand-alone programme and while a compelling evidence base exists for programmes such as GRASP^12 13^ translation into community settings, particularly in the longer term within the UK has been poor mainly due to resource restrictions.^6^ One possible way to support this is by incorporating evidence-based upper limb programmes into existing exercise programmes for stroke survivors.

One such programme is the post-rehabilitation enablement programme (PREP), based on the established Exercise Training after Stroke study.^29^ Community stroke teams have the option to refer stroke survivors to this programme on completion of statuary services community rehabilitation if there is an ongoing rehabilitation need. PREP provides a group based 60 min exercise programme once per week, combined with a 60 min health education session delivered by a local charity. Exercises within the programme focus on cardiovascular health and lower limb strengthening performed at intensity.^1^ There is no specific task-orientated, repetitive practice provision for the upper limb, and especially lacks fine motor movements as recommended in the evidence. Uptake to the programme is however high, with over 1000 participants completing the programme since it is introduction in 2018. While data is not published, outcome measures are recorded by the charity have demonstrated positive changes in mobility and quality of life measures. It therefore provides a platform to provide an evidence-based upper limb programme to stroke survivors within the community setting.

### Aim and objectives

Our study aimed to evaluate the feasibility of embedding the GRASP group-based intervention in addition to PREP alongside an established group-based exercise programme for stroke survivors as a method of enabling more people living with stroke to access evidence-based upper limb rehabilitation in the longer term. Based on a feasibility study design, our objectives were (1) to evaluate the acceptability and demand of the intervention and trial procedures placed on stroke survivors, (2) to identify adaptions within the intervention to optimise its design and (3) to determine the resource requirements to deliver the intervention. The preliminary effects of the intervention on upper limb function were also explored.

## Methods

The study design was a mixed method, cluster feasibility-controlled trial. For this study, a cluster is defined as one intervention site and one control site being delivered simultaneously.^30^

A sample size justification is outlined in the protocol to reflect this feasibility design (estimate n=60). One intervention and one control site were selected by the care coordinator, for the charity provider Northern Ireland Chest Heart and Stroke (NICHS), based on resources available to deliver a face-to-face intervention in the aftermath of the COVID-19 pandemic. The first PREP group to commence after COVID-19 was allocated as the intervention site. The subsequent group to commence was allocated as the control site. Both sites remained the intervention and control for the study period (12 months). The NICHS care coordinator informed the therapist at the PREP site if they were an intervention (PREP Plus) or control (PREP only) site.

### Recruitment

If stroke survivors have an ongoing need for rehabilitation, they are invited to attend the PREP after being discharged from NHS services. Consequently, the participants in this study were those who had already been referred to the PREP. As a result, stroke survivors were allocated to their local PREP site by family support workers before being informed of the research study occurring at that site. If service users did not want to or were ineligible to participate in the study, they were able to attend the allocated PREP site to complete the exercise session. Details of those interested in the study were passed to the research associate (RA) who provided verbal information via telephone and if interested, provisionally screened stroke survivors for eligibility. An information sheet was sent via post if they met the study inclusion criteria which included adults age 18+ with a diagnosis of first-time stroke who have completed statutory rehabilitation and are medically fit to participate in an exercise programme. Participants were required to have an impairment with their upper limb either by self or practitioner report (no restriction placed on minimum or maximum impairment level). Participants could not participate if they had a self-reported pain score of 5/more in their impaired upper limb.

### Process

Each cluster consisted of one intervention site and one control site, with each delivering the Prep Plus (intervention) or Prep only (control) across a 6-week period. There were five clusters in total, delivered in succession. All sites were rural however they had participants who travelled from both rural and urban areas to attend. Qualitative information from participants and therapists from the intervention group was used to refine the design for the next cluster. Both the intervention and control were delivered by a neurological specialist physiotherapist at a community location. The intervention therapist remained the same throughout all five clusters to prevent confounding factors, while the control group therapist changed throughout (as per normal practice).

### Training of intervention therapists

Physiotherapists attended one, 4-hour online training session with JJE, the founder of GRASP. The training introduced physiotherapists to GRASP content and delivery materials. The training focused on implementing GRASP in a group setting and provided therapists with a detailed implementation manual. Therapists attended a follow-up session with the principal investigator (KP) to clarify any intervention queries. Therapists additionally completed training with the RA (NK) focusing on the trial procedures and implementation guidance. The RA performed a fidelity check using an Apriori checklist on week 3 at the intervention site. Ongoing support was provided to the physiotherapists by the research team as needed.

### Control group

Participants attend PREP once per week for 6 weeks. This was a 2-hour session with the first 60 min consisting of 60 min of circuit-based exercises followed by a social and education session (60 min).^30^ While the exercise circuit did not include any fine motor skill practice, one gross-motor upper extremity strengthening exercise was completed within the circuit.

### Intervention group

After informed consent by the RA, the RA ascertained the level of upper limb impairment from participants (mild, moderate or severe) based on guidance used within other research protocols.^31^ This enabled the research team to prepare therapy bags correlating to the participants’ impairment level and enabled the intervention therapist to have an overview of the participants before the initial session.

The format of the initial intervention group (cluster 1) is described here however based on focus groups completed with participants and an individual interview with the therapist at the end of the first cluster, the intervention was subsequently refined (see the Results section).

Participants were required to attend a 2.5-hour session, once per week, for 6 weeks. The additional 30 min from the control group was a group-based discussion relating to GRASP guided by the therapist (table 1), after which they completed PREP, the same circuit-based exercises (60 min) and social and education sessions (60 min) as the control group.

**Table 1:** Participant demographics

	Intervention(n=26)	Control(n=35)	Overall(n=61)
Age: (years) mean (range)	61(32–80)	70(41–88)	66(32–88)
Gender (M:F)	11:15	24:11	35:26
Time post-stroke (weeks) mean (range)	38.97(4.8–160)	56.48(8.0–324.0)	49.02(4.86–324.00)
Mobility level			
No aid required	13	16	29
Walking aid required	9	13	22
Wheelchair	4	4	8
Upper limb impairment			
Mild	11	26	37
Moderate	10	5	15
Severe	5	4	9
Care received			
None	5	15	20
Family	21	20	41
Private	0	0	0
Comorbidities			
None	12	9	21
One	3	19	22
Two or more	11	7	19
Cognitive impairments			
No	14	25	39
One or more	12	10	22
Baseline clinical measures			
TUG (seconds) mean and range	18.97(6.12–62)	17.00(0–75)	17.84(0–75)
10MWT (seconds) mean and range	17.18(6.82–70)	13.60 (0–38)	15.13 (0–70)
Shortened Warwick Edinburgh (metric score) mean and range	22.43 (16.36–32.55)	24.18 (16.88–35)	23.43 (16.36–35)
REACH (score 0–5)			
0	5	2	7
1	2	2	4
2	3	2	5
3	9	4	13
4	6	9	15
5	1	16	17

10MWT10-metre walk testREACHRating of Everyday Arm-use in the Community and HomeTUGtimed get up and go

Additionally, participants were encouraged to complete 60 min of a HEP per day, as part of GRASP, which adds over 300 upper limb gross motor and fine motor skill repetitions^18^ (table 2). The two additional components to the control group were the group-based discussion (GRASP) and HEP.

**Table 2:** Weekly content

Week	In session	HEP
1	Handbook review Behavioural contract Goal setting Select and discuss appropriate exercises	Therapist identifies exercises suitable for each participant Additionally, participants encouraged to try any exercises within the range identified
2–5	Review goals Problem solving based on HEP completion Exercises adaption Education on UL daily use	Participant encouraged to problem solve based on HEP and UL daily use during previous week Patient encouraged to review link between patient goals, exercises and UL daily use
6	Review of goals Education on UL beyond programme	Encourage continuation of exercises and UL daily use beyond programme

HEPhome exercise programmeULupper limb

The content of the group-based discussion relating to GRASP followed guidance from the GRASP protocol.^20 21^ The content of the discussion varied depending on the week of the programme (table 2).

## Evaluation

The feasibility and acceptability of the intervention and trial demands were quantified by variables quantifying recruitment rates (sites and individual participants), extent of participation and attrition, resource implications (eg, therapist time) as well as by qualitative feedback gained from intervention participants (n=18 participants across five focus groups). Individual interviews were completed with the intervention therapists at the end of each cluster, focused on identifying any changes required (table 3). Focus groups were also conducted with intervention participants on the final day of the intervention by an independent researcher.

**Table 3:** Intervention changes throughout the study

Time point	Changes to subsequent intervention group	Rationale
Postintervention delivery group 1	GRASP completed before PREP: intervention participants initially completed GRASP during social time after PREP but changed to the intervention group completing this in the 30 min before PREP started	Avoid missing social aspect that all participants report being beneficial
	In session time allocated to GRASP was increased from 30 min to 45 min	Problem solving required more time to fully discuss and generate solutions
	Participant generated goals reduced from three goals to two	Goal setting was challenging for participants therefore a move to weekly goals supported generation of goals
	Text reminders to participants introduced to encourage HEP completion	Text reminders sent out for homework reminders
	HEP booklet adapted to include section for writing goals and logging exercise completion	To support participants in remembering goals and exercises completed
Postintervention delivery group 2	Participants encouraged to consider goals prior to attending	Provided additional time for problem solving
	HEP booklets adapted—dividers added, booklet reorganised	To increase ease of use and to help encourage use of HEP
Postintervention delivery group 3	Therapist notified of UL impairment level prior to session 1	Help prepare therapist and increase efficiency of problem solving
Postintervention delivery groups 4 and 5	No changes	

GRASPGraded Repetitive Arm Supplementary ProgrammeHEPhome exercise programmePREPpost-rehabilitation enablement programmeREACHRating of Everyday Arm-use in the Community and HomeULupper limb

Clinical outcomes were conducted by an independent outcome assessor (NK), blind to the site allocation. The primary clinical outcome was the Rating of Everyday Arm-use in the Community and Home (REACH).^32^ The REACH is a 6-level (0–5) classification scale that captures how the upper limb is used in everyday life. A change of one level on the scale is considered a clinically important difference.^32^ While this is a self-report measure, some participants required support from the outcome assessor to complete it. Secondary measurements were used based on outcome measures used within the current PREP, outside of the research study including the 10-metre walk test (10MWT), timed get up and go (TUG) and the Shortened Edinburgh Warwick Questionnaire.^33^

Resource requirements were only calculated for additional costs associated with the research and therefore resources required to deliver the standard PREP were not considered. Calculated based on costs identified from monthly salaries (inclusive of overheads) of the physiotherapists and RA. Equipment costs were based on purchase invoices and travel costs were calculated based on the remuneration rate of 45.5p per mile.

## Data analysis

Quantitative data were coded and entered SPSS (IBM SPSS Statistics V.28). Data entry was independently assessed for accuracy and analysed as per protocol. Intention to treat analysis was applied.

A descriptive analysis was completed to address the primary feasibility objectives and reported using means, range and percentages. This included recruitment and retention rates and baseline demographics. Only data from participants who attended 50% of sessions or more was used. The primary and secondary outcome measure analysis was completed by a statistician. As this was a feasibility study, mean difference (SD) was estimated at each follow-up time point for all outcome measures. Data is presented as mean (SD) and percentages for all clinical outcome measures apart from the REACH, which was analysed descriptively. Data for all participants who scored 5 on the REACH score was excluded from the analysis due to the ceiling effect.

Qualitative data from therapist interviews and focus groups were transcribed verbatim, coded and thematically analysed.^34^ These themes were used to inform intervention made to the next cluster.

## Patient and public involvement

Service users and stakeholders were actively involved in all stages of this research. In terms of identifying the need for a large-scale study with stroke survivors in Northern Ireland, conducted by a coapplicant (NCK) identified upper limb rehabilitation as a key priority for stroke survivors, informing the rationale for this study. This was reiterated by therapists working in the PREP. In terms of the intervention design, the PREP services coordinator and therapists from the programme outlined appropriate recruitment and intervention delivery strategies, considering resource requirements. Service users (stroke survivor/therapist) specifically explored the burden being placed on participants and identified any potential barriers to participation. During the study, a project management group, involving a patient with stroke, carer and healthcare professionals ensured smooth delivery and supported trouble shooting of any issues arising. Participants from the study have supported the development of a lay summary of the study results and coproduced a video outlining their experience.

## Results

**Figure 1:**
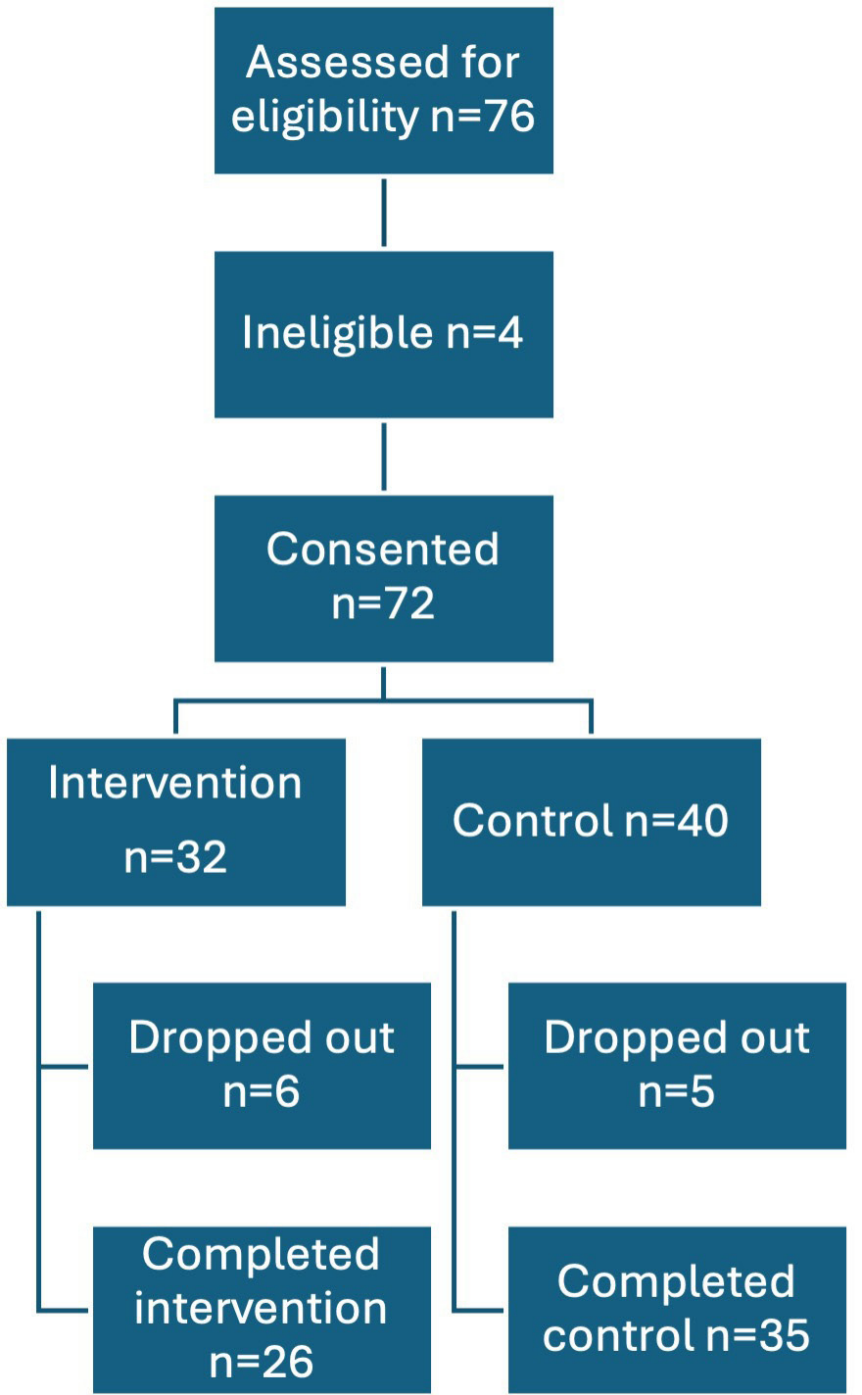
Flow of participants through the study.

The flow of participants through the study is shown in figure 1. 76 individuals were assessed for eligibility between January 2022 and October 2022 (10 months). The recruitment ended once the target sample was reached. 72 consented to be part of the study; 32 were recruited into the intervention group and 40 into the control group (figure 1).

### Participant demographics

Participants were a mean (SD) of 66 years^13^ old and 49.02 weeks post-stroke. The control group was older than the intervention group (average 8.4 years older), included more male participants and was slightly longer post-stroke (average 17.51 weeks) (table 1) however none of these differences were significant.

At the commencement of the study, most participants had mild impairment of their upper limb (n=37, 61%) and the majority had one or more comorbidities for example, living with arthritis (n=41, 67%). 22 participants (36%) reported they had mild cognitive impairment, with most requiring care support from family members (n=41, 67%). Clinically, both groups were similar at baseline concerning the 10MWT, TUG and Shortened Warwick Edinburgh. The control group had a greater proportion of participants with a REACH score of 4–5 (n=25) (indicating a higher level of function) compared with the intervention group (n=7).

### Acceptability and demand (objective 1)

99 service users attended the intervention and control sites during the study period. From these 99, the majority (n=76, 77%) were potentially eligible, indicating the high prevalence of people with stroke who identify as having an issue with their upper limb. From this, there was a high recruitment rate of 72/77 (94%) and after gaining consent and being informed which arm of the study the participant was in, no dropouts occurred. This demonstrates a high demand for the intervention from stroke survivors living in the community.

11 participants failed to complete at least 50% of the sessions. Within the control group (n=5), reasons were not related to the intervention, for example, illness (n=3) and return to work (n=2). In the intervention group, three participants dropped out with reasons related to the intervention including being too high functioning (n=1), the exercise booklet being too complicated (n=1) and too busy to complete HEP (n=1). An additional three dropouts included one participant passing away before starting the study, lack of transportation (n=1) and illness (n=1); these three participants did not start the intervention. The attrition rate was 15% which is below what is expected for stroke exercise studies,^12^ demonstrating a high acceptability of the intervention. Their demographics are included in online supplemental file 1 and are reflective of those who completed the intervention.

Focus groups were completed with each intervention group to determine the intervention acceptability of delivering GRASP alongside PREP and to identify intervention amendments. All intervention participants were invited however only 28 participated across five focus groups. All participants found GRASP to be a positive experience, with most indicating they would continue with it. Some participants identified the HEP as being challenging, influenced by other life commitments and levels of fatigue. This was noted as a consideration to outline at the outset with intervention participants. Some participants required motivational and practical support from their families to complete the programme, which for some was unexpected. Some participants reported becoming frustrated when they could not complete an exercise however, they were determined to complete the programme. Goal setting was embraced by participants to help link GRASP to everyday activities they wanted to achieve in their daily lives. Other aspects reflecting acceptability included the group setting which enhanced the social aspect, learning from others with similar experiences and problem solving together in addition to seeing the real-life impact of the programme. Participants reported the recruitment and consent processes as being positive with no changes suggested to the trial procedures. Participants were asked about outcome measurement completion and provided brief positive experiences. An overview of the main themes with quotes is outlined in online supplemental file 2.

#### Fidelity

To further explore the acceptability, one fidelity assessment was completed per intervention site (n=5 total) on week 3, using the GRASP fidelity checklist.^23^ Fidelity of the intervention was high (average 16.2/18), ranging from 15/18 (clusters 1 and 2) to 17/18 for clusters 3–5. The most common items missed included group discussion due to the therapists being required to complete individual problem-solving with participants and lack of discussion on repetitions of exercises.

### Iterative design for an optimal intervention (objective 2)

Individual interviews (n=5), completed with the intervention therapist at the end of each cluster led to the refinement of the intervention (table 3).

#### Resource requirements (objective 3)

Each intervention site cost an additional average of £920 to deliver and £225 to deliver the control site. Overall delivery costs were £5725 for five intervention and five control sites (table 4).

**Table 4:** Resource requirements

	Per intervention site(additional costs)	Per control site (additional costs)	Overall(n=5 intervention sites, 5 control sites) (£)
Staff training	£300	0	1500
Physiotherapist	£60 per hour×0.5 hours per week×1 week=£30, increased to 0.75 hours×5 weeks=£225. Total=£255	0	1275
Consumables	£140 (including printing manuals, purchasing beanbags, wrist weights, putty, paper clips and grip dynamometers)	0	700
Research associate time for outcome measurement	£24 per hour×6 hours=£144	£24 per hour×6 hours=£144	1440
Research associate travel	£27 (average) per return visit×3 visits=£81	£27 (average) per return visit×3 visits=£81	810
	£920	£225	5725

### Preliminary effects on clinical outcomes (objective 4)

#### Upper limb

Within the intervention group, most participants (n=17/26, 65%) improved by at least one level on the REACH scale. In comparison, most participants in the control group reported no change (n=26/33, 79%). No participants declined in their upper limb function within either group (table 5).

**Table 5:** Clinical outcomes

Change in score	InterventionChange from baseline to postintervention (n=25)	ControlChange from baseline to postintervention (n=19)
**REACH**	**N=25**	**N=19**
Decreased by one level	0 (0%)	0 (0%)
No change	8 (32%)	12 (63.1%)
Improved one level	13 (52%)	6 (31.6 %)
Improved two levels	4 (16%)	1 (5.2%)
**10MWT**	**N=26**	**N=35**
	3.09 (4.35)	−0.45 (16.49)
**TUG**	**N=26**	**N=35**
	3.38 (5.29)	1.23 (10.78)
**Shortened Warwick Edinburgh (metric**)	**N=26**	**N=35**
	−1.922 (4.16)	−0.25 (4.13)

* Note all participants who scored 5 on the REACH at baseline were removed from the analysis due to a ceiling effect.

10MWT10-metre walk testREACHRating of Everyday Arm-use in the Community and HomeTUGtimed get up and go

#### Mobility and quality of life measures

TUG scores improved across both groups from baseline to postintervention (intervention group; 3.38 s, control group; 1.23 s). This difference reached the MCID (Minimal Clinically Important Difference) (2.9 s) for the intervention group. The intervention group also demonstrated a greater improvement in the 10MWT (3.09 s).

Both groups demonstrated improvement in quality of life, as measured by the Shortened Warwick Edinburgh scale however this did not meet the MCID for this measure for either group (3 points).

No adverse events were reported.

## Discussion

This is the first known study which has aimed to embed the GRASP group-based intervention alongside PREP, an established community-based stroke rehabilitation programme.

The study was deemed as acceptable and in high demand demonstrated by the successful recruitment and retention rates of stroke survivors living in the community setting. Recruitment rates were higher than previous studies in stroke rehabilitation^35^ with the success being attributed to both the strong collaboration with the third-sector provider and the supportive mechanisms in place for participants. Despite the high level of commitment required from stroke survivors to complete the programme, the intervention was successfully delivered with high acceptability of the intervention demonstrated in both retention data and qualitative feedback. With no further intervention refinements being noted after cluster 4 and 5, it can be determined that the process of optimising the intervention design through qualitative methods, was successful in designing and intervention which was acceptable to both participants and the intervention therapist. Within the intervention group participants demonstrated an improvement in their upper limb function demonstrating the potential impact of this intervention on community-based stroke survivors.

The feasibility of delivery and acceptability of the intervention to the participants, as demonstrated in table 3 and the qualitative data, can be attributed to both the iterative design process and the inbuilt behaviour change mechanisms. The coproduction of the research with community providers and other stakeholders from the onset also added to this success. GRASP is built on behaviour change concepts^12^ including the incorporation of goal setting and the use of an exercise log, aligning to the COM-B model of behaviour change.^36^ However, the further behaviour change mechanisms added, for example, text message reminders to reinforce behaviour, added to this intervention’s success, as reported in focus groups by participants. The role of the physiotherapist as a coach also became apparent during the intervention, supporting behaviour change. In a traditional medical model, the physiotherapist takes on a direct role, providing treatments. During this intervention, the therapists reported in their individual interviews that they collaborated with and supported the patient, empowering them to take an active role in their rehabilitation. This mechanism supported service users to engage in all aspects of the intervention, fostering positive outcomes. This collaborative approach has been reflected in other successful upper limb-based therapies^37^ and should be considered an approach for rehabilitation moving forward.

Using an iterative design, driven by participant feedback, ensured the intervention was suitable for the end user.^38^ Within this study, the collective expertise of both stroke survivors and therapists delivering the intervention resulted in a patient-centred approach to intervention design, as demonstrated in table 3. The codesign approach to design the GRASP and PREP intervention delivery methods ensured any challenges with intervention implementation were addressed early, resulting in positive outcomes. This streamlined development approach also enabled the intervention to be personalised to the population and setting (community-based stroke survivors). The final iteration of the intervention reflects a sustainable option for long-term delivery due to the codesign approach adopted.

Collaboration with a charity and voluntary organisation was key to the establishment and delivery of this study. The organisation was responsible for selecting the study site and participants and maintaining open communication with the research team throughout the study. The importance of such collaborative relationships has also been recognised by other studies that have collaborated with third sector organisations.^39 40^ Recruiting from an established programme, PREP, provided benefits to support recruitment and retaining participants within the programme. Like other group based programmes, the added social value gained from being in a group setting, enhances the likelihood of retention.^41^,^43^

While evidence suggests approximately 50% of stroke survivors have an upper limb impairment at 6 months^8^ in our study 75% of stroke survivors using the PREP service identified themselves as having an impairment of their upper limb. This may in part be attributed to the self-report nature of the upper limb criteria used for the study. It therefore raises the question as to whether more stroke survivors recognise incomplete recovery of their upper limb than therapists or clinical measures identify. While participants with mild impairment of the upper limb are often excluded from studies, future studies should consider their inclusion based on self-report impact on stroke survivors.

Participants demonstrated significant differences across a range of clinical measures in comparison to the control group, as outlined in table 4. Of interest this also applied to mobility measures—while the additional intervention (GRASP) did not focus on mobility, this does suggest a link. This link between mobility and upper limb recovery has been explored in other studies^35^ questioning the need to add task-orientated, repetitive upper limb exercises into generic exercise programmes for stroke survivors, to support improvements in mobility and further outcome measures to investigate this. Furthermore, participants within the control group, which did not focus on upper limb rehabilitation, noted upper limb improvement for seven participants. This suggests that general exercise can improve upper limb function, possibly related to stroke survivors being more active after completing general exercise and therefore impacting the amount of use of the upper limb or if overall physical conditioning has a carry-over to the upper limb. Future studies should explore this link through use of accurate outcome measurement.

While improvements were noted in quality of life across both groups, which could be attributed to several factors such as the social and educational aspect of the group setting and improvement across clinical measures, the intervention group demonstrated a greater improvement. This indicates a potential link between improved upper limb function and overall quality of life, an aspect which should be measured going forward to further explore this relationship.

A strength of this study was the use of the same therapist at all intervention sites throughout the entire study as it enabled the therapist to become familiar with the intervention and provide experienced feedback after each iteration. Having participants with a range of upper limb impairment levels enabled comparison across impairment levels. This study aimed to recruit 60 participants, yet 72 participants were recruited, and 61 participants completed the study. This successful recruitment and low attrition rate give us insight into the acceptability of the programme. Potential limitations included the choice of outcome measures used, a more established or routinely used arm and hand clinical measure could have been used. Additionally, the impact of COVID-19 and the reduction in the number of sites available at the start of the study resulted in the inability to complete site randomisation. Furthermore, the resource implications to deliver the intervention were not costed using health economics approaches. Therefore, additional costs such as RA support to the intervention sites were not calculated; future studies should consider a full economic costing.

## Conclusion

This study successfully recruited and retained stroke survivors into an upper limb community-based programme. It poses a feasible delivery mechanism to combine evidence-based upper limb approaches with established physical activity programmes. The intervention resulted in positive changes across a range of clinical measures, demonstrating its potential impact. Sufficient learning has been made to inform a future large scale randomised controlled trial.

## supplementary material

10.1136/bmjopen-2024-088301 online supplemental file 1

10.1136/bmjopen-2024-088301 online supplemental file 2

## Data Availability

Data are available upon reasonable request.
