# Supplementary material for: Post-rehabilitation programme to support upper limb recovery in community-dwelling stroke survivors: a mixed methods cluster-feasibility controlled trial
Source: BMJ Open. 2024 Oct 15;14(10):e088301. doi: 10.1136/bmjopen-2024-088301 (PMC11481143; doi:10.1136/bmjopen-2024-088301)
Supplement: online supplemental file 1 [file bmjopen-14-10-s001.pdf]

Demographic overview for participants who dropped out

	**Intervention (n=6)**	**Control (n=5)**
**Age**: [y] mean (range)	64 (51-75)	66 (44-78)
**Gender** (M: F)	4:2	3:3
**Time post stroke** [weeks] mean (range)	27 (8-39)	122 (18-421)
No aid required	5	5
Walking aid required	1	1
Wheelchair	0	0
Upper limb impairment		
Mild	5	5
Moderate	1	0
Severe	0	0
**Care received**		
None	4	2
Family	1	1
Private	1	2
**Co morbidities**		
None	2	3
One	2	2
Two or more	2	0
**Cognitive impairments**		
No	5	5
One or more	1	0
**Baseline clinical measures**		
TUG [seconds] mean and range	11.9 (8.36-20.37)	12.69 (9.08-19.85)
10MWT [seconds] mean and range	10.20 (8.08-15.71)	10.62 (7.8-13.26)
Shortened Warwick Edinburgh [metric score] mean and range	23.25 (15-34)	27.3 (19-33)
REACH (score 0-5)		
0	0	0
1	0	0
2	2	0
3	1	1
4	2	2
5	1	2
