# Supplementary material for: Post-rehabilitation programme to support upper limb recovery in community-dwelling stroke survivors: a mixed methods cluster-feasibility controlled trial
Source: BMJ Open. 2024 Oct 15;14(10):e088301. doi: 10.1136/bmjopen-2024-088301 (PMC11481143; doi:10.1136/bmjopen-2024-088301)
Supplement: online supplemental file 2 [file bmjopen-14-10-s002.pdf]

Themes with supporting quotes from participant focus groups

Theme	Quote examples
Deployment Dynamics<br><br>*Participants discussed different methods of ensuring they completed their home exercise programme to ensure it was completed.*	P7, FG2: on some days I did 40 minutes and other weeks up to the hour<br><br>P15, FG5: This is our hour, And no matter what happens. Even if the doorbell rings, I sit there.<br><br>P17 FG5: The time scale every day, you know it's the 20 minutes or 15 minutes is enough for me.<br><br>P3, FG1: It's very simple. It doesn't need that equipment. We can do it at home so that's good.<br><br>P7, FG2: Then spread between the two sections and doing 30 minutes on 30 minutes was easier better<br><br>P12, FG3: But on occasions, what you were doing in new, actually, you weren't really sure if you were doing it correctly or you could have been impinging<br><br>P18, FG5: The book is fine. I had no problems with that.<br><br>P11, FG3: At the beginning I was anxious about the whole thing…Then as it went on I realized I could do the exercises and could do more than I thought I could.
Empowerment Essentials<br><br>*For some participants they required help to complete the programme, whilst others were fully independent.*	P12, FG3; I needed A little bit of help here (pointing to fingers)<br><br>P8, FG2: My husband was there sitting on the other side of the bench and encouraging me. We are retired so, we're quite happy to work away.<br><br>P3, FG1: I was totally independent. I'm not very good at somebody hanging over my shoulder so I sort of am sort of self-disciplined so I got into a routine of doing it<br><br>P13, FG4: Sometimes I needed help just with that hand . But that's it really.
Community connection<br><br>*For many, the community aspect of a group setting was empowering and supported them beyond upper limb rehabilitation.*	P1, FG1: It gets you out of the house to meet people that has the same problems.<br><br>P12, FG3: I like the social aspect too. It is better to do something here than, by yourself at home.<br><br>P16, FG5: Yeah, you see, with other people there, the comradery and all..<br><br>P14, FG4: I like the social aspect too<br><br>P13, FG4: I said the social aspect will be good for me because I am by myself with my cat

	P4, FG1: Staying at home, sometimes, people forget to exercise
Physical Progress<br><br>*Many examples were provided for specific everyday tasks that people were able to complete as a result of the programme*.	P3, FG1: Trying to incorporate day to day tasks, like so, I made one of my goals for a week was to put my dishes away using my left hand, instead of my right that was always instinctively used.<br><br>P12, FG4: My goal was to button my jumper and I could not do it. I have to take off all always the jumper is and big button. But this morning, I was able to button on my jumper.<br><br>P15, FG4: Husband speaking about wife (participant): She could never hold her hand straight, the fingers are always closed so for her to hold something small like a pen, was impossible. Today now, the fingers are out straight. She actually can use a pen.<br><br>P10, FG3: This is only after a few weeks of working that business of exercising the fingers and there been enough a difference. It really has been made that big a difference.<br><br>P14, FG5: Whenever I get my breakfast I'm told to put butter and marmalade on toast and it used to just sit there, I couldn't do it…. I'm actually putting it on this last couple mornings with my left hand.<br>I think mentally it's good for you as well
Forward Focus<br><br>*All participants indicated they would try to continue with the programme due to their progress and the ability to complete it at home.*	P3, FG1: I would certainly continue with it, because I can see the benefits now.<br><br>P15, FG5: We intend to keep us going because now I am on the road to is recovery, it's much better than I was.<br><br>P4, FG3: I think repeating all the exercises going to be good because it's it's very simple. It doesn't need that equipment. We can do it at home so that's it.<br><br>P15, FG5: I want to recover a wee bit more, I don't want to give up anymore for my daughter.
