# Supplementary material for: Post-rehabilitation programme to support upper limb recovery in community-dwelling stroke survivors: a mixed methods cluster-feasibility controlled trial
Source: BMJ Open. 2024 Oct 15;14(10):e088301. doi: 10.1136/bmjopen-2024-088301 (PMC11481143; doi:10.1136/bmjopen-2024-088301)
Supplement: Supplementary file 3 [file bmjopen-2024-088301.reviewer_comments.pdf]

PEER REVIEW HISTORY

BMJ Open publishes all reviews undertaken for accepted manuscripts. Reviewers are asked to complete a checklist review form and are provided with free text boxes to elaborate on their assessment. These free text comments are reproduced below.

ARTICLE DETAILS

Title (Provisional)

A post-rehabilitation programme to support upper limb recovery in community-dwelling stroke survivors; A mixed methods cluster-feasibility controlled trial.

Authors

pedlow, katy; Kennedy, Niamh C; Klempel, Natalie; Eng, Janice J; Adamson, Gary; Hylands, Jenny; Hughes, Noelene; Campbell, Zoe; McDonough, Suzanne

---

VERSION 1 - REVIEW

**Reviewer**	**1**
**Name**	**Kumar, Praveen**
**Affiliation**	**University of the West of England**
**Date**	**28-Jun-2024**
**COI**	**I have worked with one of the authors on a grant application but that project is unrelated to one under review.**

PREP Plus feasibility study; A post-rehabilitation programme to support upper limb recovery in community-dwelling stroke survivors.

Thank you for giving opportunity to review this paper. Upper limb (UL) impairment affects 80% of stroke survivors. The updated national clinical guidelines for stroke in the UK (2023) recommend that PwS should receive a minimum of 3 hours of therapy per day, 5 days per week, both in hospital and at home,with renewed focus on UL rehabilitation provision. This is an interesting work but requires substantial revisions before it can be published. I provide my comments below:

Title: This needs to be clearer. Avoid starting with Abbreviation in the title. PREP should be written in full. Unsure what PREP plus is? Re-think and present the title clearly highlighting the intervention, and design of the study clearly.

Abstract:

Background is too vague. It is good to highlight the UL problems, but this needs to be done succinctly. Here you need to introduce / provide critical information on your proposed interventions that you are testing as part of this study. You introduce both PREP and GRASP

in Methods section, instead, brief introduction plus evidence of these two should be provided here. There is an assumption that reader has knowledge and evidence linked to these interventions. Also, I cannot find clear aim and objectives presented at the end of this section. These should then be clearly linked to methods. What feasibility aspects did you intend to address in this study?

Methods: If this was controlled trial, this should be reflected in the title. What was the sample size estimate? How many were you intending to recruit? Sixty-one…… belongs to results section. Did participants receive PREP and GRASP in addition to standard care? What was the purpose of mixed methods? Was the refined intervention tested? Or was the purpose of this trial to refine to inform future trial? What outcomes were tested for? Did you use any clinical outcome measures?

Results: It will be good to see some numbers % regarding recruitment and retention. It would be good to present results clearly – what was the demographic distribution – numbers, age, gender in each group, descriptive analysis of the results, clinical outcomes. I can find any qualitative results being reported here.

Conclusion: Does the study provide evidence to carry out full trial in the future.

Overall, abstract lacks relevant information and does not provide sufficient information to the reader about the study aims, purpose, methods, results and conclusion. Information provided is vague and lacks scientific rigour.

Strength and limitations of the study

This section should highlight key strengths and weaknesses rather than repeating the aim and methods, results. This section should provide clear messages relating to strengths and weaknesses. I appreciate comment around COVID, however what were other limitations? Was there issue around blind assessment? Or from participants feedback, were there any challenges with using the intervention etc.

I see authors use the terms 'acceptability and demand of combining two interventions' this is new information and should be introduced in the background section of the abstract.

Background:

There is some relevant information however, majority of information lack's structure and flow. It would be good keep the focus on UL problems, facts and figures, what are the recommended interventions?

Huang J, Ji JR, Liang C, et al. Effects of physical therapy-based rehabilitation on recovery of upper limb motor function after stroke in adults: a systematic review and meta-analysis of randomized controlled trials. Ann Palliat Med. 2022;11(2):521-531. doi:10.21037/apm-21-3710

Tenberg S, Mueller S, Vogt L, et al. Comparative Effectiveness of Upper Limb Exercise Interventions in Individuals With Stroke: A Network Meta-Analysis. Stroke. 2023;54(7):1839-1853. doi:10.1161/STROKEAHA.123.043110.

Also CIMT is recommended in clinical guidelines, so how your proposed intervention offers something beneficial and different to this established programme. Some critical discussion would be helpful.

Some critical discussion on GRASP and PREP is required. What are the challenges of implementing GRASP in community, why long-term implementation had been a problem. Very limited information is provided on PREP in the last paragraph. More information and evidence, critical discussion is required. There is reference to community-based group programme. Reading in the Methods and Results section, it appears that PREP is usual care provided in the region. What is the evidence of effectiveness as you have included this as a control group?

Aims: It is unclear how GRASP will be embedded and if PREP is a group-based programme? Also authors have used PREP plus in the title but there is no reference to 'plus' here. It is confusing if authors are embedding GRASP into PREP or vice-versa. The aim needs to be clearly stated explaining what the intervention the study is testing for feasibility? Whether GRASP is being tested for feasibility along with PREP compared to PREP only or the other way round? What about usual care?

There are few studies cited by the authors but the critical analysis of literature is unclear and so is the rationale. You need to provide clear background information, what are the problems, why are they relevant? The intervention selected? Why is this important, advantageous of the intervention. Overall, background section needs to be re-written with evidence of critical analysis of the literature, identifying clear gaps leading to clear aims and objectives of the study.

Methods: Cluster needs to be defined and explained at the outset. Selection of sites and how many clusters were selected? Over what period of time? As highlighted before, PREP plus and PREP only should be introduced and defined clearly in the aims.

Recruitment section – This is not clear to me. Authors say, if participants were not eligible, they were allocated to PREP programme? Is PREP provided as part of standard care? This is very confusing as I am not sure if PREP is standard care? Also, it is unclear at what point after stroke where participants recruited into the study? Did you consider random allocation?

Training: Was any additional training or resources provided to the therapists? What was covered in training on implementation guidance?

Intervention group: In addition to GRASP, participants also received additional HEP. This is new information provided here. I think, the intervention / purpose should be made clearer at the outset.

The information provided on intervention – PREP / GRASP/ HEP is very confusing to follow as authors have muddled the information provided and it is difficult to keep a track of what is being offered to the participants. The structure of information and logical flow needs to be improved all the way through.

Evaluation: It is unclear what was evaluated through interviews and focus groups? As this information was used to refine the intervention for subsequent clusters, it is unclear whether this was done and how subsequent sessions reflected those modifications. That needs to be included in the intervention section.

Outcomes: it is unclear why 10-meter walk and TUG were included. I cannot fin any other UL related outcome measure except REACH.

Data analysis: Did you screen for eligibility? How many were screened, and how many were recruited?

Patient and Public Involvement: This is important, but not sure if it is required under separate heading in a paper for publication. This is more relevant for a grant application. I wonder if this information could be added succinctly in the background section as it helped to inform the study aims/methods etc.

Results: Age should be presented as whole number without decimals and there is repetition of this information in the results section. It will be helpful to have clear presentation of results. Alike my previous comments, please present this section with key results using appropriate paragraphs or sub-headings. It is difficult to keep a track of findings due to lack of focus. Also, why only 18 participants were interviewed? Where all these participants from the intervention group or also included participants from PREP only group.

Iterative Design: it is unclear why cluster one had so many changes. I thought, cluster one was delivered as originally planned and changes were made to subsequent clusters.

Discussion: Start with re-stating the aim of your study followed by key results. Then discuss your key findings relating to the objectives of your study. You need to discuss the recruitment and retention rates, are these acceptable when compared to other studies. What were the advantages of recruiting from the known groups? Does this lead to high recruitment and retention rates? Did role of third sector organisation play a role in increasing recruitment and retention? More critical discussion by comparing and contrasting with the existing evidence is required.

Conclusion: Did this feasibility study provide sufficient information for conducting future trial?

Reviewer 2

Name Langhammer, Birgitta
Affiliation Oslo Metropolitan University, Department of Physiotherapy
Date 29-Jun-2024
COI no competing interests

Comments bmjopen-2024-088301

Title: PREP Plus feasibility study; A post-rehabilitation programme to support upper limb recovery in community dwelling stroke survivors

The title is a bit misleading, in the methods you state you use a mixed methods so as a reader I expect a bit more than just "feasibility".

General comments:

The study has as an aim to

(1) To evaluate the acceptability and demand of the intervention and trial procedures placed on stroke survivors and their carers,

To include arm training in a post stroke programme should not be considered more complex? Individual needs for rehab should be at focus according to guidelines…

As for the acceptability and demands placed on stroke survivors and their carers, this is not presented in depth?

(2) To identify adaptions within the intervention to optimise its design

This is not clearly described- it is mentioned that changes were made " as the study went on" but as a reader I do not see what these changes were?

and (3) To determine the resource requirements to deliver the intervention. To analyse…

By this I understand a view of how much extra personnel, time is needed. It is not clearly presented either? This could have been an interesting point.

Abstract:

Background:

"substantial problem" seems a bit vague- please specify how big is the problem

what is meant with "recovery"? better performance ?

do you mean feasible to include arm training???

and what exactly is meant with acceptable 1from the range of stakeholders ?

p 2 Strengths and limitations: needs to be rewritten in view of the aim…

why could the randomization of clusters not take place because of COVID?

Methods:

The first sentence needs to be more detailed “The study design was a mixed method, cluster feasibility-controlled trial” - What was what?

Recruitment:

Were the participants first time stroke?

How much was the impairment of the arm?

Process:

“clusters”- Were different communities involved? Rural / urban ?

Refinement of the intervention for the next cluster? is this not part of the aim to investigate and present?

P5 time is longer in the intervention group this can be confounding factor...

P6: who did the evaluations of the clinical outcomes?

was REACH self evaluation?

Why were the data on the best performers excluded? It is not logical…

P 7: “ During the study a project management group, involving a patient with stroke, carer and healthcare professionals ensured smooth delivery and supported trouble shooting of any issues arising”

it seems this was not a random allocation?

to what extent did this influence the results?

Results:

P7 that means 15% drop out...why did they drop out?

Table 2: do you have any data on the drop outs?

P9 “ a small number”… I would call this "drop out"..

As for acceptability: was it not the whole programme PREP+GRASP that was to be investigated vs PREP only?

P 10 “upper limb” - an alternative outcome for arm and hand had been of value to explore the extent of the intervention

P 12 l10: ? one would think that any stroke victime with impairment of the arm and hand would be happy to participate, a welcome addition to rehabilitation? Why did you think this was not feasible?

L 19 How was behavior change evaluated?

L 23: a mismatch? you just stated that text messages was required to maintain behavior?

L 26: this is referral to CIMT which is not that straight forward....see requirements for eligibility

and time to do the exercises while constrained...

l34: the co-design … do you mean the PREP therapy?

L 43 I am not familiar with the term third sector? what is that?

L 52: you mention another number in your introduction...

L 58-59: I think another outcome measure would narrow the number down?

P 13 l29-31: it is not stated anywhere how many sites you intended to include? If the power calculation was 60 patients then it was sufficient was it not?

However I did not see how you did the power in the methods?

Another thing you state mixed methods, in what way did you use that? It is not clear from this paper?

Conclusion:

L 31 was it first time stroke? we know little of their baseline function in terms of objective measurements.

Do you mean there is a division of functionalities in the rehabilitation post stroke? Is not arm and hand training evaluated on the same premises as mobility?

**Reviewer**	**3**
**Name**	**Luo, Zhiqiang**
**Affiliation**	**Foshan University, SCHOOL OF DESIGN**
**Date**	**01-Jul-2024**
**COI**	**NO**

The study is significant given the long-term rehabilitation needs for stroke survivors in the community, where they lack the same effective rehabilitation practices as in hospitals. The study explores a comprehensive approach to support stroke survivors' rehabilitation in the community. Thus, the manuscript could be recommended for acceptance with some revisions, see the attached documents.

The present manuscript describes a feasibility study to embed upper limb programs, the Graded Repetitive Arm Supplementary Program (GRASP), into established community-based physical activity programs, the Post Rehabilitation Enablement Program (PREP). The study compared the participants' performances in terms of qualitative and quantitative data under two groups: the control group (PREP) and the intervention group (PREP+GRASP). The results not only posed a feasible delivery mechanism to combine the two programs, but also demonstrated a greater improvement in the intervention group within the REACH upper limb measure and the 10MWT.

The study is significant given the long-term rehabilitation needs for stroke survivors in the community, where they lack the same effective rehabilitation practices as in hospitals. The study explores a comprehensive approach to support stroke survivors' rehabilitation in the community.

Thus, the manuscript could be recommended for acceptance after emphasizing the following points:

1. Ensure that each claim made in the Discussion is directly linked to the data presented in the Results. This strengthens the argument and provides clear evidence for each conclusion drawn. For example, the first sentence of the second paragraph on page 12, "The feasibility of delivery and acceptability of intervention to the participants can be attributed to both the iterative design process and the inbuilt behaviour change", could refer to the results reported in Table 3 as well as the qualitative feedback.

2. Extent the Discussion to emphasis on Result. For example, elaborate on the findings in Table 4, providing possible explanations for observed changes. Discuss potential factors influencing these outcomes and their implications for practice

3. Regarding the iterative design approach, include a detailed analysis of the performance differences among the clusters in the Results section, especially the performance differences among Cluster 1, 2, and 3. This will provide a clearer picture of how iterative changes impacted participant outcomes

4. Highlight key themes from qualitative feedback, providing direct quotes, if possible, to give depth to the participants' experiences and perceptions.

VERSION 1 - AUTHOR RESPONSE

Please see responses to reviewers in the attached file

Comment	Response
Reviewer: 1	
Title: This needs to be clearer. Avoid starting with Abbreviation in the title. PREP should be written in full. Unsure what PREP plus is? Re-think and present the title clearly highlighting the intervention, and design of the study clearly.	A new title has been amended to 'A post-rehabilitation programme to support upper limb recovery in community-dwelling stroke survivors; A mixed methods cluster-feasibility controlled trial.
Abstract - Background is too vague. It is good to highlight the UL problems, but this needs to be done succinctly. Here you need to introduce / provide critical information on your proposed interventions that you are testing as part of this study. You introduce both PREP and GRASP in Methods section, instead, brief	The background has been revisited to provide succinct information on the proposed interventions. The aim and objectives have enhanced at the bottom of this section.

introduction plus evidence of these two should be provided here. There is an assumption that reader has knowledge and evidence linked to these interventions. Also, I cannot find clear aim and objectives presented at the end of this section. These should then be clearly linked to methods. What feasibility aspects did you intend to address in this study?	
Methods: If this was controlled trial, this should be reflected in the title. What was the sample size estimate? How many were you intending to recruit? Sixty-one…… belongs to results section. Did participants receive PREP and GRASP in addition to standard care? What was the purpose of mixed methods? Was the refined intervention tested? Or was the purpose of this trial to refine to inform future trial? What outcomes were tested for? Did you use any clinical outcome measures?	The title has now been adapted.<br>The estimate sample size (n=60) has been added into the main study methods.<br>Participants who were not receiving standard care has been added to this section. The purpose of mixed-methods was to ensure that participants and therapists experience and views could be incorporated into the next iteration of the intervention.<br>The refined intervention was tested at each stage before being refined for the following stage. By iteration 4 there were no further suggestions to intervention design changes.<br>Outcomes have also been added to this methods section in abstract and evaluation section of Methods.
Results: It will be good to see some numbers % regarding recruitment and retention. It would be good to present results clearly – what was the demographic distribution – numbers, age, gender in each group, descriptive analysis of the results, clinical outcomes. I can find any qualitative results being reported here.	Numbers / % have been added for recruitment and retention. Some basic demographic information has also been added. Brief qualitative results are now reported in the abstract.
Conclusion: Does the study provide evidence to carry out full trial in the future.<br>Overall, abstract lacks relevant information and does not provide sufficient information to the reader about the study aims, purpose, methods, results and conclusion. Information provided is vague and lacks scientific rigour.	The following has been added to the conclusion; in a future large scale and fully powered study.
Strength and limitations of the study<br>This section should highlight key strengths and weaknesses rather than repeating the aim and methods, results. This section should provide clear messages relating to strengths and weaknesses. I appreciate comment around COVID, however what were other limitations? Was there issue	The strengths and limitations section has been reviewed and revised to provide a clear message about the strengths and weaknesses.<br>The updated background section of the abstract reflects ‘acceptability and demand of combining two interventions.’

around blind assessment? Or from participants feedback, were there any challenges with using the intervention etc. I see authors use the terms 'acceptability and demand of combining two interventions' this is new information and should be introduced in the background section of the abstract.	
Background	
There is some relevant information however, majority of information lack's structure and flow. It would be good keep the focus on UL problems, facts and figures, what are the recommended interventions?<br>Huang J, Ji JR, Liang C, et al. Effects of physical therapy-based rehabilitation on recovery of upper limb motor function after stroke in adults: a systematic review and meta-analysis of randomized controlled trials. Ann Palliat Med. 2022;11(2):521-531. doi:10.21037/apm-21-3710<br>Tenberg S, Mueller S, Vogt L, et al. Comparative Effectiveness of Upper Limb Exercise Interventions in Individuals With Stroke: A Network Meta-Analysis. Stroke. 2023;54(7):1839-1853. doi:10.1161/STROKEAHA.123.043110.	This section has been revised to ensure it is more succinct and provides greater clarity on the current evidence base and recommendations. Thank you for highlighting these references – they are now included in the background.
Also CIMT is recommended in clinical guidelines, so how your proposed intervention offers something beneficial and different to this established programme. Some critical discussion would be helpful.	The following has been added; Grounded by similar neuroplasticity principles and behaviour change aspects as constraint induced movement therapy, such as high repetition, goal setting and education [11, 14, 15, 16, 17] the lack of constraint may make GRASP more amenable to community dwelling adults.
Some critical discussion on GRASP and PREP is required. What are the challenges of implementing GRASP in community, why long-term implementation had been a problem. Very limited information is provided on PREP in the last paragraph. More information and evidence, critical discussion is required. There is reference to community-based group programme. Reading in the Methods and Results section, it appears that PREP is usual care provided in the region. What is the evidence of effectiveness as you have included this as a control group?	Additional critique has been added throughout this section such as 'GRASP has traditionally been delivered as a stand-alone programme and whilst a compelling evidence base exists for programmes such as GRASP [12,13] translation into community settings, particularly in the longer-term within the UK has been poor mainly due to resource restrictions[6].'<br>PREP is not usual care in the region – this has been clarified with rewording in the background.

Aims: It is unclear how GRASP will be embedded and if PREP is a group-based programme? Also authors have used PREP plus in the title but there is no reference to 'plus' here. It is confusing if authors are embedding GRASP into PREP or vice-versa. The aim needs to be clearly stated explaining what the intervention the study is testing for feasibility? Whether GRASP is being tested for feasibility along with PREP compared to PREP only or the other way round? What about usual care?	The term 'alongside' has replaced 'into' to clarify that GRASP was to be delivered alongside the PREP programme.<br>Group based has also been added.<br>The title is now changed to not include the term PREP.<br>The following has been added to clarify that it is testing the feasibility of delivering GRASP alongside PREP 'as a method of enabling more people living with stroke to access evidence based upper limb rehabilitation in the longer term.
There are few studies cited by the authors but the critical analysis of literature is unclear and so is the rationale. You need to provide clear background information, what are the problems, why are they relevant? The intervention selected? Why is this important, advantageous of the intervention. Overall, background section needs to be re-written with evidence of critical analysis of the literature, identifying clear gaps leading to clear aims and objectives of the study.	The background has now been reviewed and rewritten.
Methods: Cluster needs to be defined and explained at the outset. Selection of sites and how many clusters were selected? Over what period of time? As highlighted before, PREP plus and PREP only should be introduced and defined clearly in the aims.<br>Recruitment section – This is not clear to me. Authors say, if participants were not eligible, they were allocated to PREP programme? Is PREP provided as part of standard care? This is very confusing as I am not sure if PREP is standard care? Also, it is unclear at what point after stroke where participants recruited into the study? Did you consider random allocation?	The following has been added to the start of the methods 'For this study a cluster is defined as one intervention site and one control site being delivered simultaneously.'<br>It has been clarified in this section there was one intervention and one control site over a 12 month study period.<br>The aims have been revised for clarity.<br>Recruitment; The following has been added 'Stroke survivors are offered to attend PREP once their community rehabilitation is completed, if there is an ongoing rehabilitation need. The purposeful sample for this study were therefore participants who had already been referred to attend the PREP programme.'
Training: Was any additional training or resources provided to the therapists? What was covered in training on implementation guidance?<br>Intervention group: In addition to GRASP, participants also received additional HEP. This is new information provided here. I think, the intervention / purpose should be made clearer at the outset.	The following has been added to the training section; Therapists attended a follow up session with the PI to clarify any intervention queries.<br>The following has also been added 'The training focused on implementing GRASP in a group setting and provided therapists with a detailed implementation manual.'<br>HEP is now added to the intervention overview in the background section

The information provided on intervention – PREP / GRASP/ HEP is very confusing to follow as authors have muddled the information provided and it is difficult to keep a track of what is being offered to the participants. The structure of information and logical flow needs to be improved all the way through.	The description of the control and intervention group have been revised in addition to the background section to ensure they are reflective of each other.
Evaluation: It is unclear what was evaluated through interviews and focus groups? As this information was used to refine the intervention for subsequent clusters, it is unclear whether this was done and how subsequent sessions reflected those modifications. That needs to be included in the intervention section.	The following has been added to the intervention 'The format of the initial intervention group (cluster one) is described here however based on focus groups completed with participants and an individual interview with the therapist at the end of the first cluster, the intervention was subsequently refined (see results section).'
Outcomes: it is unclear why 10-meter walk and TUG were included. I cannot fin any other UL related outcome measure except REACH.	This has been reworded for clarity within the evaluation section; Secondary measurements were used based on outcome measures used within the current PREP, outside of the research study including; the10-meter walk test (10MWT), Timed get up and go (TUG) and the Shortened Edinburgh Warwick Questionnaire [28]. Note that the REACH was recommended for us to use by the team who created GRASP. '
Data analysis: Did you screen for eligibility? How many were screened, and how many were recruited?	This is included in the results section
Patient and Public Involvement: This is important, but not sure if it is required under separate heading in a paper for publication. This is more relevant for a grant application. I wonder if this information could be added succinctly in the background section as it helped to inform the study aims/methods etc.* *editor's note: The journal's format requires a Patient and Public Involvement statement with its own heading.	As per editor guidance the PPI section is included as a separate section.
Results: Age should be presented as whole number without decimals and there is repetition of this information in the results section. It will be helpful to have clear presentation of results. Alike my previous comments, please present this section with key results using appropriate paragraphs or sub-headings. It is difficult to keep a track of findings due to lack of focus.	The decimals have now been removed and this section has been reviewed for clarity.

Also, why only 18 participants were interviewed? Where all these participants from the intervention group or also included participants from PREP only group.	This was a typo – 28 participants completed focus groups. This has also been clarified 'Focus groups were completed with each intervention group participants to determine the intervention acceptability and to identify intervention amendments. All participants were invited however only 28 participated across 5 focus groups.'
Iterative Design: it is unclear why cluster one had so many changes. I thought, cluster one was delivered as originally planned and changes were made to subsequent clusters.	Apologies this was confusing- cluster one was delivered as planned and then the initial suggestions were made by cluster one participants to inform cluster 2 etc. The wording has been amended in the table to reflect this.
Discussion: Start with re-stating the aim of your study followed by key results. Then discuss your key findings relating to the objectives of your study. You need to discuss the recruitment and retention rates, are these acceptable when compared to other studies. What were the advantages of recruiting from the known groups? Does this lead to high recruitment and retention rates? Did role of third sector organisation play a role in increasing recruitment and retention? More critical discussion by comparing and contrasting with the existing evidence is required.	The aim of the study has been added alongside a summary of key findings.
Conclusion: Did this feasibility study provide sufficient information for conducting future trial?	The following has been added; Sufficient learning has been made to inform a future large scale randomised controlled trial.
Reviewer: 2	
The title is a bit misleading, in the methods you state you use a mixed methods so as a reader I expect a bit more than just "feasibility".	The title has been reworded to 'A post-rehabilitation programme to support upper limb recovery in community-dwelling stroke survivors; A mixed methods cluster-feasibility controlled trial.
The study has as an aim to (1) To evaluate the acceptability and demand of the intervention and trial procedures placed on stroke survivors and their carers,<br>To include arm training in a post stroke programme should not be considered more complex? Individual needs for rehab should be at focus according to guidelines…	The complexity for this study is knowing the logistics of how best to deliver the programme in this setting, especially considering aspects such as fatigue etc. It is also considering optimising resources and enabling access to specific rehab in the longer term. The background has been revised and is now hopefully more clear.
As for the acceptability and demands placed on stroke survivors and their carers, this is not presented in depth?	'And carers' has been removed as this was written in error and was not explored.

	Within ‘Acceptability and demand’ in the results section additional details have been added in red to add depth to this section. In addition, a supplementary file has been added with additional detail on focus group analysis.
(2) To identify adaptions within the intervention to optimise its design<br>This is not clearly described- it is mentioned that changes were made “ as the study went on” but as a reader I do not see what these changes were?<br>and	Table three outlines the changes made to the intervention- additional references to this has now been made throughout the document.
(3) To determine the resource requirements to deliver the intervention. To analyse…<br>By this I understand a view of how much extra personnel, time is needed. It is not clearly presented either? This could have been an interesting point.	A resource table has been added to the results section, however there are some limitations with this which have been noted within the discussion.
Abstract:<br>Background:<br>"substantial problem" seems a bit vague-please specify how big is the problem	This has been changed to ‘Less than 15% of stroke survivors regain their pre-stroke level of function in their upper limb, compounded with a lack of long-term rehabilitation options available to improve use of their arm and hand.’
what is meant with "recovery"? better performance ?	This has been changed to ‘improve use of their arm and hand.’
do you mean feasible to include arm training???<br>and what exactly is meant with acceptable 1from the range of stakeholders ?	This has been reworded to ‘We aimed to establish if this was feasible to deliver and what the experience of stroke survivors and therapists were in completing the intervention, including any refinements required to the intervention design and the acceptability of the trial procedures.’
p 2 Strengths and limitations: needs to be rewritten in view of the aim…<br>why could the randomization of clusters not take place because of COVID?	These have been rewritten to reflect the aim in addition to clarity being added re: Covid-19.
Methods; The first sentence needs to be more detailed “The study design was a mixed method, cluster feasibility-controlled trial” - What was what?	This has been reworded to ‘A cluster feasibility-controlled trial was conducted using a both quantitative and qualitative measures.’
Recruitment:<br>Were the participants first time stroke?	‘First time’ has been added to the inclusion criteria.
How much was the impairment of the arm?	No restriction placed on minimum or maximum impairment level has been added to the inclusion criteria.
Process:<br>“clusters”- Were different communities	This has been added to process ‘All sites were rural however they had participants

involved? Rural / urban ?	who travelled from both rural and urban areas to attend.'
Refinement of the intervention for the next cluster? is this not part of the aim to investigate and present?	Yes this was part of the aims of the study and the results of this process are presented in the results section.
P5 time is longer in the intervention group this can be confounding factor...	The 30 minutes extra in the intervention group was to complete the GRASP discussion. This section has been refined and hopefully this is more clear to the reader.
P6: who did the evaluations of the clinical outcomes?	(NK) has been added for clarity.
was REACH self evaluation?<br>Why were the data on the best performers excluded? It is not logical…	The following has been added 'Whilst this is a self report measure, some participants required support from the outcome assessor to complete it.'
Why were the data on the best performers excluded? It is not logical…	If they scored 5 at baseline, they could not improve any on the REACH scale and therefore were excluded for this measure only.
Results:<br>P7 that means 15% drop out...why did they drop out?	This has been explored within the section 'acceptability and demand' and therefore removed from this initial results paragraph
Table 2: do you have any data on the drop outs?	This has been added to a new file called supplementary file 1. A line has also been added to the results section.
P9 " a small number"… I would call this "drop out"..	This has been reworded for clarity.
As for acceptability: was it not the whole programme PREP+GRASP that was to be investigated vs PREP only?	This has been reworded for clarity
P 10 "upper limb" - an alternative outcome for arm and hand had been of value to explore the extent of the intervention	Agree- this has been added to the limitations within the discussion.
P 12 l10: ? one would think that any stroke victime with impairment of the arm and hand would be happy to participate, a welcome addition to rehabilitation? Why did you think this was not feasible?	The following line has been added 'Despite the high level of commitment required from stroke survivors to complete the programme.'
L 19 How was behavior change evaluated?	The following has been added 'as reported in focus groups by participants.'
L 23: a mismatch? you just stated that text messages was required to maintain behavior?	This line was misleading and has therefore been reworded to use the terms 'added to'.
L 26: this is referral to CIMT which is not that straight forward....see requirements for eligibility<br>and time to do the exercises while constrained...	This sentence has been refined to highlight that it is the collaborative approach which was successfully used in other interventions such as CIMT.
l34: the co-design … do you mean the PREP therapy?	'To design the GRASP and PREP intervention delivery methods' has been added for clarity.

L 43 I am not familiar with the term third sector? what is that?	This has been replaced with 'charity and voluntary'.
L 52: you mention another number in your introduction...	This was misleading and therefore the introduction has been changed to be reflective of the upper limb. 50% is the correct number.
L 58-59: I think another outcome measure would narrow the number down?	'and further outcome measures to investigate this' has been added.
P 13 l29-31: it is not stated anywhere how many sites you intended to include? If the power calculation was 60 patients then it was sufficient was it not? However I did not see how you did the power in the methods?	The power calculation of 60 has been added to the methods for clarity.
Another thing you state mixed methods, in what way did you use that? It is not clear from this paper?	Mixed methods refers to the use of both qualitative and quantitative methods.
Conclusion:<br>L 31 was it first time stroke? we know little of their baseline function in terms of objective measurements.	First time stroke has been added to the participant information.<br>Baseline clinical measures are included in table 2.
Do you mean there is a division of functionalities in the rehabilitation post stroke? Is not arm and hand training evaluated on the same premises as mobility?	This sentence has been refined for clarity, now referring to 'positive changes.'
Reviewer: 3	
Ensure that each claim made in the Discussion is directly linked to the data presented in the Results. This strengthens the argument and provides clear evidence for each conclusion drawn. For example, the first sentence of the second paragraph on page 12, "The feasibility of delivery and acceptability of intervention to the participants can be attributed to both the iterative design process and the inbuilt behaviour change", could refer to the results reported in Table 3 as well as the qualitative feedback.	The discussion has been reviewed to make a number of direct links back to the presented data.
Extent the Discussion to emphasis on Result. For example, elaborate on the findings in Table 4, providing possible explanations for observed changes. Discuss<br>potential factors influencing these outcomes and their implications for practice	This has been elaborated throughout the discussion, for example the following has been added 'Furthermore, participants within the control group, which did not focus on upper limb rehabilitation, noted upper limb improvement for seven participants. This suggests that general exercise can improve upper limb function, possibly related to stroke survivors being more active after completing general exercise and therefore impacting the amount of use of the upper limb or if overall physical conditioning has a carry over to the

	upper limb. Future studies should explore this link through use of accurate outcome measurement.'
Regarding the iterative design approach, include a detailed analysis of the performance differences among the clusters in the Results section, especially the<br>performance differences among Cluster 1, 2, and 3. This will provide a clearer picture of how iterative changes impacted participant outcomes	This was our original plan and we had sought advice from our statistician in regards to analysing the data using this methods. However due to the small sample size within each cluster and the feasibility nature of the study, it was advised not to present the results by cluster as they would not be meaningful.
4. Highlight key themes from qualitative feedback, providing direct quotes, if possible,<br>to give depth to the participants' experiences and perceptions.	A supplementary table has now been added to provide additional depth to the participants experience.

VERSION 2 - REVIEW

**Reviewer**	**1**
**Name**	**Kumar, Praveen**
**Affiliation**	**University of the West of England**
**Date**	**11-Sep-2024**
**COI**	**None**

Authors have done a great job of addressing the comments raised the reviewers. However, there are few outstanding issues that needs to be addressed before the paper can be accepted for publication. I appreciate the authors had to add relevant information based on the comments raised by the reviewers, the information needs to be presented succinctly. The word count for the paper is now 4000 plus words as suggested by authors. Wonder if this can be reduced and unsure how it fits with publisher guidelines.

Abstract- This is 407 words now. Suggest reduce it and present the information succinctly.

Methods:

"Participants who were not receiving standard care, completed PREP for 6 weeks (control), with the intervention group also completing GRASP."

If I interpret this correctly, patients who were discharged from the usual NHS service were recruited into this study. And one group completed PREP only and the intervention group received PREP Plus GRASP. If this is correct, please amend this.

The following sentence seems to be incomplete to me.

Grounded by similar neuroplasticity principles and behaviour change aspects as constraint induced movement therapy, such as high repetition, goal setting and education [12, 14, 18, 19, 20, 21].

Please reword these statement:

One potential option to support this, is the embedding of evidence based upper limb programs into established exercise programmes for stroke survivors. which are well established.

One such programme is the post-rehabilitation enablement program [PREP] based on the established Exercise Training after Stroke study (REF).

Aims and Objectives:

As you have now introduced PREP in the previous paragraphs, you could re-word this

Our study aimed to evaluate the feasibility of embedding the GRASP group-based intervention in addition to PREP alongside an established group-based exercise programme for stroke survivors as a method of enabling more people living with stroke to access evidence based upper limb rehabilitation in the longer term.

Recruitment

Please re-word, re-structure this sentence for clarity

If there is an ongoing rehabilitation need, stroke survivors are offered to attend PREP once they are discharged from the NHS/community rehabilitation services is completed,. The purposeful sample for this study were therefore participants who had already been referred to attend the PREP programme.

Discussion – Add PREP to the following sentence

This is the first known study which has aimed to embed the GRASP group-based intervention alongside PREP an established community-based stroke rehabilitation program.

VERSION 2 - AUTHOR RESPONSE

Many thanks to the reviewers for providing valuable suggestions to improve the overall quality of this paper. These have now been addressed within the manuscript.

We look forward to your next correspondence

Comment	Response
Abstract- This is 407 words now. Suggest reduce it and present the information succinctly.	This has now been reviewed to make it more succinct; it is now 353 words.

Methods:<br>"Participants who were not receiving standard care, completed PREP for 6 weeks (control), with the intervention group also completing GRASP."<br>If I interpret this correctly, patients who were discharged from the usual NHS service were recruited into this study. And one group completed PREP only and the intervention group received PREP Plus GRASP. If this is correct, please amend this.	The following has been added to the methods of the abstract; who were discharged from NHS care.
The following sentence seems to be incomplete to me.<br>Grounded by similar neuroplasticity principles and behaviour change aspects as constraint induced movement therapy, such as high repetition, goal setting and education [12, 14, 18, 19, 20, 21].	This sentence has now been revised to 'The programme is grounded by neuroplasticity principles and behaviour change aspects similar to constraint induced movement therapy, including high repetition, goal setting and education.'
Please reword these statement:<br>One potential option to support this, is the embedding of evidence based upper limb programs into established exercise programmes for stroke survivors. which are well established. One such programme is the post-rehabilitation enablement program [PREP] based on the established Exercise Training after Stroke study (REF).	The first statement has been reworded to 'One possible way to support this is by incorporating evidence-based upper limb programs into existing exercise programs for stroke survivors'
Aims and Objectives:<br><br>As you have now introduced PREP in the previous paragraphs, you could re-word this<br>Our study aimed to evaluate the feasibility of embedding the GRASP group-based intervention in addition to PREP alongside an established group-based exercise programme for stroke survivors as a method of enabling more people living with stroke to access evidence based upper limb rehabilitation in the longer term.	This has now been re-worded as suggested.
Recruitment<br>Please re-word, re-structure this sentence for clarity<br>If there is an ongoing rehabilitation need, stroke survivors are offered to attend PREP once they are discharged from the NHS/community rehabilitation services is completed,. The purposeful sample for this study were therefore	This has been reworded to;<br>If stroke survivors have an ongoing need for rehabilitation, they are invited to attend the PREP program after being discharged from NHS services. Consequently, the participants in this study were those who had

participants who had already been referred to attend the PREP programme.	already been referred to the PREP programme.
Discussion – Add PREP to the following sentence This is the first known study which has aimed to embed the GRASP group-based intervention alongside PREP an established community-based stroke rehabilitation program.	This has now been added as suggested
